# Kikuchi-Fujimoto Disease in a Young African American Male

**DOI:** 10.7759/cureus.2508

**Published:** 2018-04-19

**Authors:** Ahmed Zaghloul, Corina Iorgoveanu, Andrew Polio, Aakash Desai

**Affiliations:** 1 Internal Medicine, University of Connecticut Health Center, Farmington, USA

**Keywords:** kikuchi-fujimoto disease, histiocytic necrotizing lymphadenitis

## Abstract

Kikuchi-Fujimoto disease (KFD), also known as histiocytic necrotizing lymphadenitis, is an extremely rare, self-limiting disorder which typically presents with fever and painful, unilateral cervical lymphadenopathy in previously healthy individuals. Here, we describe a case of KFD which initially presented with fever of unknown origin. Due to its non-specific symptoms and low incidence, KFD poses a diagnostic conundrum for clinicians. Awareness of this disease entity is the key for prompt diagnosis and treatment.

## Introduction

Kikuchi-Fujimoto disease (KFD), also known as histiocytic necrotizing lymphadenitis, was originally described by Kikuchi and Fujimoto in 1972 in Japan. It is an extremely rare, self-limiting disorder which typically presents with fever and painful, unilateral cervical lymphadenopathy in previously healthy individuals. The disease is most prevalent in young East Asian or Japanese women. We describe an epidemiologically atypical patient who initially presented with fever of unknown origin and was later diagnosed with KFD. Due to its presenting symptoms, KFD is commonly mistaken for more sinister diseases, such as malignant lymphoma, tuberculosis, or adenocarcinoma. It is important to emphasize this disease entity as prompt diagnosis and treatment of the disease can help avoid unnecessary testing and discomfort for the patients.

## Case presentation

**Figure 1:**
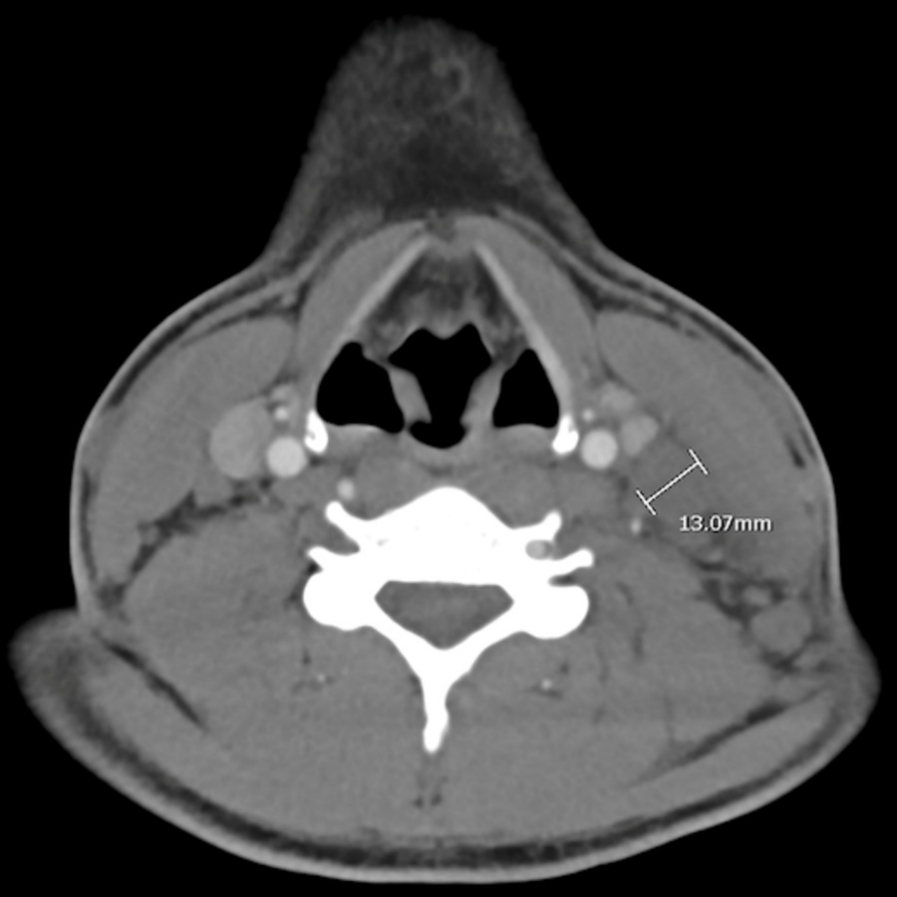
Computed tomography (CT) scan of the neck with adenopathy in the left posterior cervical carotid chain.

**Figure 2:**
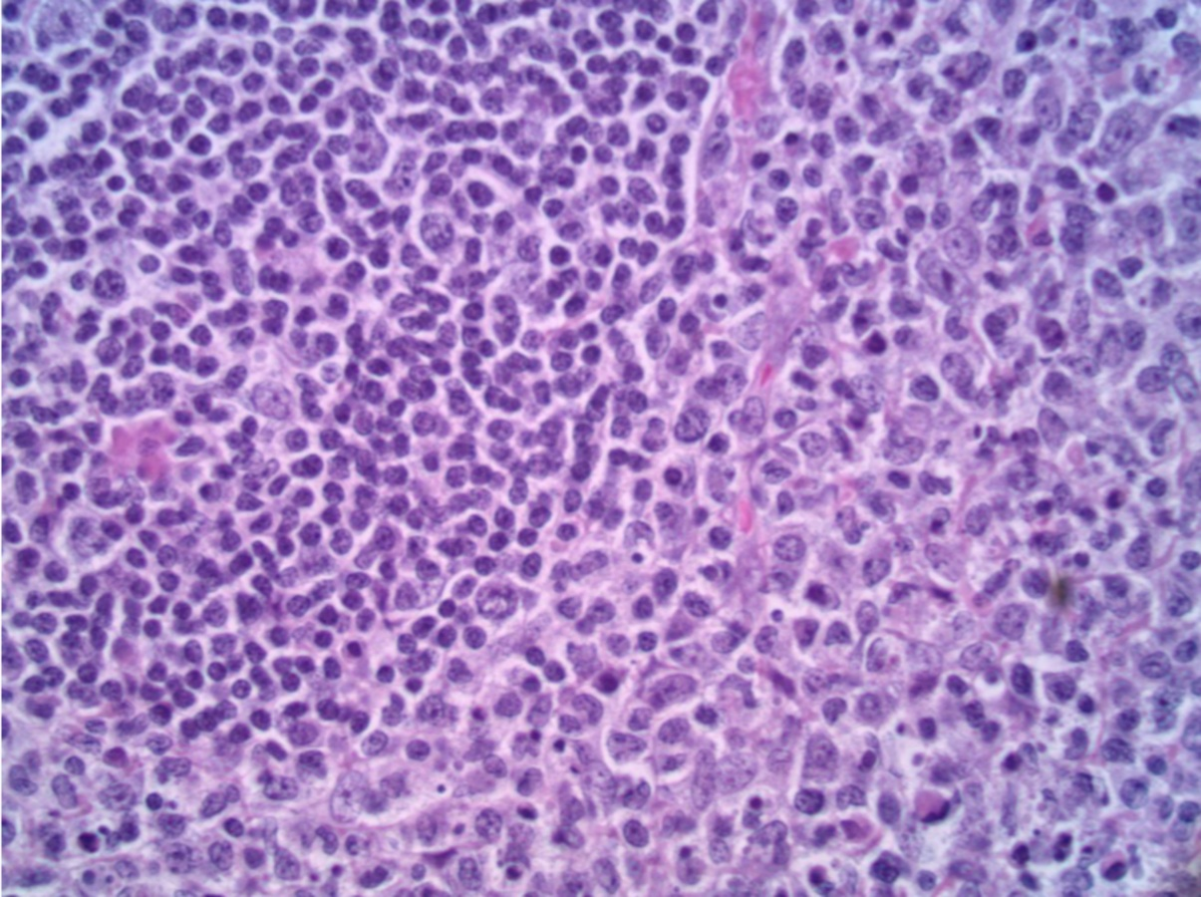
Diffuse pattern alteration of normal architecture secondary to proliferation of histiocytes associated with pyknosis or prominent karyorrhectic debris.

A 27-year-old African American male with a past medical history of asthma and allergic rhinitis presented with a three-week history fever of unknown origin. Associated symptoms included drenching night sweats, weight loss, fatigue, nausea, and diarrhea. On physical examination, he was found to be hemodynamically stable and febrile at 102°F. He was also found to have extensive left sided, posterior, cervical lymphadenopathy. Laboratory studies indicated a mild anemia with negative hepatitis panel, anti-nuclear antibody (ANA), human immunodeficiency virus (HIV1/HIV2), Lyme antibody, and infectious mononucleosis (IM) serology, non-reactive rapid plasma reagin (RPR), and a negative Babesiosis smear. Computed tomography (CT scan) of the chest and abdomen were unremarkable. Ultrasound (U/S) and CT imaging of the neck demonstrated extensive adenopathy throughout the left posterior cervical carotid chain (Figure 1). U/S guided lymph node biopsy showed polymorphous appearance with areas of zonal necrosis and a subpopulation of medium-large atypical lymphoid cells suggesting an atypical lymphoproliferative disorder. Excision biopsy was performed to rule out malignancy and Hodgkin disease. Pathology showed histiocytic necrotizing lymphadenitis (Figure 2). The histologic, immunohistochemical and flow cytometric findings were consistent with histiocytic necrotizing lymphadenitis. Immunohistochemical stains were performed to include BCL2, CD2, CD3, CD5, CD7, CD15, CD20, CD30, CD34, CD68, CD117, CD138, Epstein–Barr virus (EBV), herpes simplex virus (HSV), PAX5, Alk1, cytomegalovirus (CMV), Ki67, pankeratin, S100. Proliferating histiocytes marked with CD68 were negative for the listed T cell and B cell markers. Phenotypic analysis by flow cytometry revealed a mixed population of mature T and B lymphocytes with normal T cell:B cell ratio for lymph node. Monoclonal T cell or B cell populations were not detected and there was no immunophenotypic evidence of an acute leukemic process. The patient was managed with ibuprofen and fully recovered in five months’ time.

## Discussion

While the pathologic mechanism of KFD is unknown, it has been proposed that an immune response of T cells and histiocytes to a viral agent is responsible. While numerous offenders have been implicated including human herpes virus (HHV-6), HHV-7, HHV-8, HIV, Yersinia enterocolitica, toxoplasma gondii, and EBV, it is unclear if these are causative or coincidental factors [1-4]. Systemic lupus erythematous (SLE) has also been implicated in the pathogenesis of KFD, although whether KFD is a feature of SLE or if they are two separate entities that commonly co-exist remains to be elucidated [5]. Classically, patients present with fever and unilateral cervical lymphadenopathy, however non-specific symptoms of night sweats, nausea, vomiting, maculopapular rash and weight loss are also reported. Laboratory findings may be unrevealing, although many case reports describe leukopenia, elevated sedimentation rates, serum transaminases and low-density lipoprotein (LDL). This disorder does not have a characteristic imaging pattern, making excision biopsy of affected lymph nodes required for diagnosis. Histopathology demonstrates irregular paracortical areas of coagulative necrosis with abundant karyorrhectic debris and large numbers of different types of histiocytes at the margin of the necrotic areas. Immunohistochemical studies indicate CD8+ T cell predominance and histiocyte expression of myeloperoxidase (MPO) and CD68 [6]. Diagnostically, due to shared clinical and histological characteristics, KFD is commonly mistaken for tuberculosis, lymphoma, SLE, and, rarely, metastatic adenocarcinoma. The clinical course of KFD is self-limited with resolution in a majority of patients within one to four months. While rare, recurrence in 3-4% of patients has been reported [7]. There is no specific treatment regimen for patients with KFD and symptomatic management may be the best approach with the use of analgesics and antipyretics.

## Conclusions

KFD is a rare, self-limited necrotizing lymphadenitis with excellent prognosis and a predilection for young, Asiatic populations. While unconfirmed, recent clinical and histopathological evidence suggests a viral etiology. Recognition of this disease is vital because it may mimic lymphoma or metastatic adenocarcinoma. It is important to emphasize this disease entity as prompt diagnosis and treatment of the disease can help avoid unnecessary testing and inappropriate treatment for the patients.
